# Supplementary material for: Facile immobilization of glucose oxidase onto gold nanostars with enhanced binding affinity and optimal function
Source: R Soc Open Sci. 2019 May 1;6(5):190205. doi: 10.1098/rsos.190205 (PMC6549951; doi:10.1098/rsos.190205)
Supplement: Facile immobilisation of glucose oxidase onto gold nanostars with enhanced binding affinity and optimal function Supplementary information [file rsos190205supp1.docx]

Facile immobilisation of glucose oxidase onto gold nanostars with enhanced binding affinity and optimal function Supplementary information

Masauso Moses Phiri,* Danielle Wingrove Mulder, Shayne Mason and Barend Christiaan Vorster**

Centre for Human Metabolomics, North-West University, Potchefstroom, South Africa


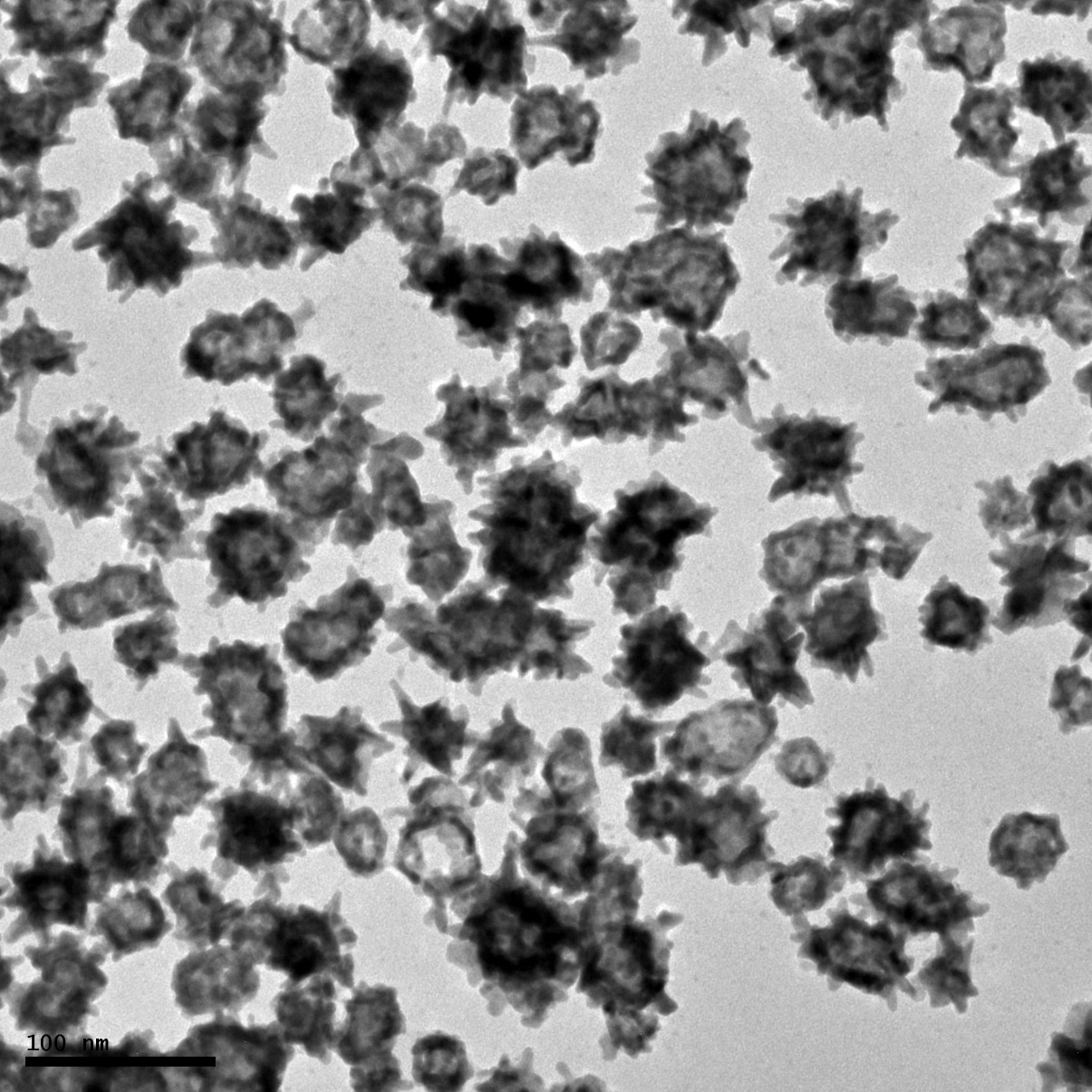


Figure 1 (Suppl) TEM image showing the AuNSs after conjugation with GOx at lower magnification.
